# Changes in temporomandibular joint anatomy, changes in condylar translation, and their relationship with disc displacement: magnetic resonance imaging study

**DOI:** 10.1590/0100-3984.2018.0020

**Published:** 2019

**Authors:** Luciane Marie Bedran, Alair Augusto Sarmet Moreira Damas dos Santos

**Affiliations:** 1 Universidade Federal Fluminense (UFF), Niterói, RJ, Brazil.

**Keywords:** Magnetic resonance imaging, Temporomandibular joint, Temporomandibular joint disc, Temporomandibular joint disorders, Ressonância magnética, Articulação temporomandibular, Disco da articulação temporomandibular, Transtornos da articulação temporomandibular

## Abstract

**Objective:**

To assess changes in the articular surfaces of the temporomandibular joint (TMJ) and in condylar translation, as detected by magnetic resonance imaging (MRI), determining whether such changes correlate with disc displacement.

**Materials and Methods:**

We retrospectively analyzed the MRI scans of 2076 TMJs of 1038 patients with symptoms of temporomandibular disorder. We attempted to determine whether articular disc deformity and changes in condylar translation, as well as changes in the articular surfaces of the condyle, glenoid fossa, and articular eminence, correlated with disc displacement.

**Results:**

Disc displacement with reduction was associated with changes in the shape of the articular eminence. Disc displacement without reduction was most strongly associated with disc deformity, condylar degeneration, glenoid fossa degeneration, and effusion. Neither decreases nor increases in condylar translation were associated with disc deformity, degenerative bone changes, or disc displacement.

**Conclusion:**

Changes in the shape of the articular eminence seem to predispose to progression of internal derangement of the TMJ.

## Abstract (pt)

**Objetivo:**

Verificar a correlação entre as alterações das superfícies articulares e da translação condilar com o deslocamento de disco da articulação temporomandibular.

**Materiais e Métodos:**

Foram analisados os exames de ressonância magnética de 2076 articulações temporomandibulares de pacientes sintomáticos de desordens temporomandibulares. A deformidade do disco articular, as alterações nas superfícies articulares do côndilo, da fossa glenoide e da eminência articular e as alterações na translação condilar foram correlacionadas com o deslocamento do disco.

**Resultados:**

O deslocamento do disco com redução demonstrou associação com as alterações de forma da eminência articular. O deslocamento do disco sem redução apresentou maior associação com a deformidade do disco, degeneração do côndilo e da fossa glenoide e efusão. Diminuição e aumento da translação condilar não apresentaram associação com a deformidade do disco, com alterações ósseas degenerativas, nem com o deslocamento do disco.

**Conclusão:**

Mudanças na forma da eminência articular parecem predispor à progressão do desarranjo interno da articulação temporomandibular.

## INTRODUCTION

Temporomandibular disorders (TMDs) are the leading cause of maxillofacial pain, involving changes in the masticatory muscles and internal derangement of the temporomandibular joint (TMJ). Internal derangement is an abnormal relationship between the articular disc, condyle, glenoid fossa, and articular eminence^(^^1^^-^^3^^)^. Anterior disc displacement is the most common disorder in patients with TMD. Posterior disc displacement, lateral disc displacement, and medial disc displacement have been described only rarely in the literature^(^^1^^,^^4^^)^.

It has been suggested that progression of disc displacement leads to degenerative changes in the disc itself and in the articular surfaces^(^^1^^,^^5^^-^^7^^)^, such changes presenting as radiological signs of osteoarthritis. Some authors have suggested that the anatomy of the eminence may predispose to disc displacement, whereas others have proposed that, conversely, disc displacement may lead to changes in the shape of the eminence^(^^1^^,^^6^^,^^8^^,^^9^^)^. There is not enough evidence to establish a relationship between changes in the glenoid fossa and internal derangement of the TMJ^(^^8^^)^. Morphological and positional abnormalities of the disc have also been associated with abnormalities in condylar translation and TMJ effusion, although this remains controversial^(^^3^^,^^10^^,^^11^^)^.

Magnetic resonance imaging (MRI), considered the gold standard for the diagnosis of head and neck abnormalities^(^^12^^-^^16^^)^, particularly TMJ changes, provides excellent contrast in soft tissues, without radiation exposure or surgical invasion, and is widely used to assess the configuration and position of the articular disc, as well as bone changes and effusion^(^^1^^,^^5^^,^^7^^,^^9^^,^^10^^,^^17^^,^^18^^)^. Pain in and dysfunction of the TMJ are common clinical problems and, according to some studies, affect up to 28% of the population^(^^19^^)^.

The aim of this study was to determine whether disc deformity, morphologic changes (in the condyle, glenoid fossa, and articular eminence), and changes in condylar translation correlate with disc displacement in patients with symptoms of TMD. The large number of cases analyzed here could provide more detailed analyses to further clarify the relationships that disc displacement has with the morphological and dynamic changes that occur in the TMJ.

## MATERIALS AND METHODS

This was a retrospective study of patients with suspected TMD who underwent MRI for between January 2007 and December 2014 at one of two centers: a private tertiary referral hospital and a private medical imaging clinic. The study was approved by the local research ethics committee (Reference no. 143-11; CAAE no. 0148.0.258.000-11).

The inclusion criterion was having reported at least one of the following signs or symptoms of TMD: pain, joint clicking, and limited range of motion. Patients were not categorized according to Angle's classification; all classes were represented in the study. Patients with a clinical history of rheumatoid arthritis, facial growth disorders, facial bone fracture, or other trauma were excluded, as were those with a history of hyperplasia, hypoplasia, malignant neoplasms of the condyle, ankylosis, or previous TMJ surgery.

A total of 1038 patients were included. All patients underwent MRI in 1.5 T scanners: Symphony (Siemens Medical Solutions, Erlangen, Germany), at the private tertiary referral hospital; or HDxt (GE Healthcare, Milwaukee, WI, USA), at the private medical imaging clinic. Sagittal and coronal images of both TMJs (n = 2076) were acquired in multiplanar T1-weighted spin echo, T2-weighted fast spin echo, proton density-weighted fat-suppressed, and gradient-echo T2*-weighted sequences, with a closed mouth and with different degrees of mouth opening-10 mm, 20 mm, and 40 mm (or the widest opening tolerated by the patient). The average execution time of the scans was 20 min. Images were read on dedicated workstations using software programs specific to each institution. The RIS/PACS platforms used were our PACs system IDCE and NET-PACS.

All images were assessed by two radiologists, each with more than 15 years of experience in MRI, who prepared the diagnostic reports according to the criteria established by Ahmad et al.^(^^20^^)^ for disc anatomy and position, bone changes, condylar translation, and effusion. The radiologists evaluated the examinations independently. In cases of disagreement, the final result was obtained by consensus. The findings were subsequently reviewed and critically analyzed by the authors.

The articular disc was considered deformed when it did not exhibit the normal biconcave configuration. The position of the articular disc was considered normal when the posterior band of the disc was located between 11 and 12 o'clock in relation to the condyle; any position between 6 o'clock and 11 o'clock was considered anterior disc displacement, whereas any position between 12 o'clock and 6 o'clock was considered posterior disc displacement.

**Figure 1:**
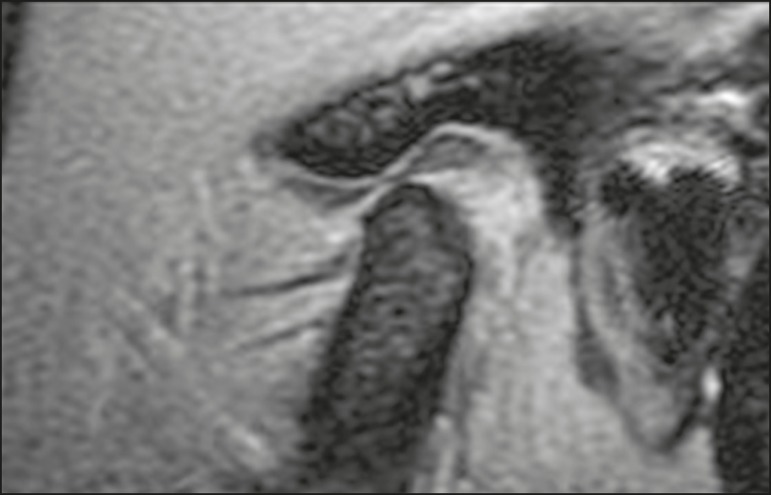
T1-weighted central sagittal MRI scan of the TMJ. In the closedmouth position, the articular disc is biconcave and in the normal position in relation to the condyle.

**Figure 2:**
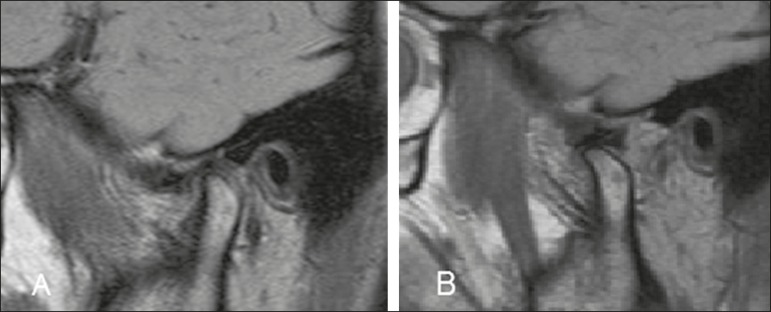
MRI showing DDWR. **A:** Closed-mouth position: articular disc in anterior position in relation to the condyle; **B:** In the open-mouth position, the condyle recaptures the dislocated disc.

**Figure 3:**
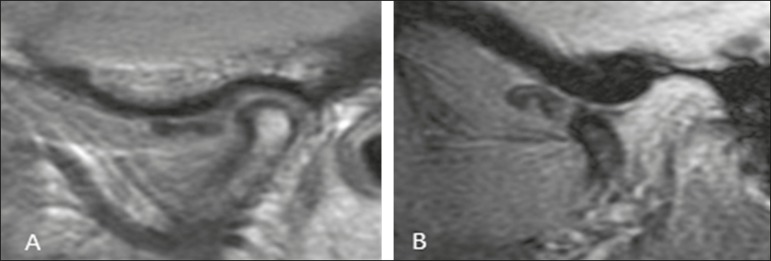
Sagittal MRI scans of the TMJ in a patient with DDWoR and disc deformity. **A:** Closed-mouth position: articular disc in anterior position in relation to the condyle; osteophyte visible in the condyle. **B:** Open-mouth position: the condyle does not reduce the dislocated disc.

On the basis of the MRI findings, the articular disc was classified as follows: normal function-disc location normal on closed- and open-mouth images (Figure 1); disc displacement with reduction (DDWR)-disc location displaced on closed-mouth images but normal on open-mouth images (Figure 2); disc displacement without reduction (DDWoR)-disc location displaced on closed- and open-mouth images (Figure 3); posterior disc displacement-posterior band of the disc clearly in contact with the bilaminar zone; lateral disc displacement-disc displaced laterally in relation to the condyle; or medial disc displacement-disc displaced medially in relation to the condyle. The condyle was considered degenerated when it exhibited osteophytes, flattening, surface erosion, a subcortical cyst, or sclerosis. Glenoid fossa degeneration and changes in the shape of the articular eminence were characterized by articular surface flattening, surface erosion, or subcortical sclerosis. Condylar translation was evaluated on open-mouth sagittal proton density-weighted MRI scans and was classified as follows: normal condylar translation-the apex of the condyle translating to the apex of the articular eminence; reduced condylar translation-the apex of the condyle translating to below the apex of the articular eminence; or increased condylar translation-the apex of the condyle translating beyond the apex of the articular eminence. The diagnosis of joint effusion was considered in the presence of hyperintense signals in the articular spaces on T2-weighted and proton density-weighted images, in open- or closed-mouth sagittal views.

### Statistical analysis

To estimate prevalence rates and assess associations, we used the chi-square test or Fisher's exact test. The measure of risk used was the odds ratio, and significance was evaluated with 95% confidence intervals. The level of statistical significance was set at 5%.

## RESULTS

The final sample comprised 1038 patients with symptoms of TMD: 891 women (85.8%) and 147 men (14.2%). Ages ranged from 16 to 85 years (mean, 38.1 years). The most well represented age group (accounting for 26.0% of the sample) was 30-39 years, followed by 20-29 years (accounting for 25.9% of the sample).

Statistical analysis of the 2076 TMJs showed that 817 (39.4%) were normal on MRI, whereas 1259 (60.7%) presented one or more types of abnormality. The main abnormalities were condyle degeneration (in 25.0%), DDWoR (in 20.6%), DDWR (in 14.2%), decreased condylar translation (in 13.3%), disc deformity (in 9.4%), posterior disc displacement (in 2.9%), increased condylar translation (in 2.3%), glenoid fossa degeneration (in 2.0%), effusion (in 1.0%), change in the shape of the articular eminence (in 0.6%), bilaminar zone degeneration (in 0.2%), lateral disc displacement (in 0.1%), and absence of translation (in 0.1%). The frequency of other changes was negligible. The associations of DDWR, DDWoR, and posterior disc displacement with the variables disc deformity, bone changes, and effusion are presented in Tables 1, 2, and 3, respectively.

**Table 1:** Associations of anterior DDWR with disc deformity, degenerative bone changes, and effusion.

	DDWR							
	Absent (n = 1781)		Present (n = 295)		Overall (n = 2076)			
Change in TMJ	N (%)		N (%)		N (%)	*P*-value	OR	95% CI
Disc morphology								
Disc deformity	151 (8.5)		44 (14.9)		195 (9.4)	0.000*	1.89	1.3-2.7
Degenerative bone changes								
Condylar degeneration	352 (19.8)		70 (23.7)		422 (20.3)	0.117*	1.3	0.9-1.7
Change in articular eminence shape	4 (0.2)		8 (2.7)		12 (0.6)	0.000*	12.4	3.7-41.4
Glenoid fossa degeneration	35 (2.0)		6 (2.0)		41 (2.0)	0.937†	1.0	0.4-2.5
Effusion	19 (1.1)		2 (0.7)		21 (1.0)	0.757†	0.6	0.1-2.7

* Chi-square test;

† Fisher's exact test. OR, odds ratio; CI, confidence interval.

**Table 2:** Associations of anterior DDWoR with disc deformity, degenerative bone changes, and effusion.

	DDWoR							
	Absent (n = 1648)		Present (n = 428)		Overall (n = 2076)			
Change in TMJ	N (%)		N (%)		N (%)	*P*-value	OR	95% CI
Disc morphology								
Disc deformity	93 (5.6)		102 (23.8)		195 (9.4)	0.000*	5.2	3.9-7.1
Degenerative bone changes								
Condylar degeneration	216 (13.1)		206 (48.1)		422 (20.3)	0.000*	6.2	4.9-7.8
Change in articular eminence shape	9 (0.5)		3 (0.7)		12 (0.6)	0.707†	1.3	0.35-4.8
Glenoid fossa degeneration	16 (1.0)		25 (5.8)		41 (2.0)	0.000†	6.3	3.3-12.0
Effusion	7 (0.4)		14 (3.3)		21 (1.0)	0.000†	7.9	3.1-19.8

* Chi-square test;

† Fisher's exact test. OR, odds ratio; CI, confidence interval.

**Table 3:** Associations of posterior disc displacement with disc deformity, degenerative bone changes, and effusion.

	Posterior disc displacement							
	Absent (n = 2016)		Present (n = 60)		Overall (n = 2076)			
Change in TMJ	N (%)		N (%)		N (%)	*P*-value	OR	95% CI
Disc morphology								
Disc deformity	178 (8.8)		17 (28.3)		195 (9.4)	0.000*	4.08	2.3-7.3
Degenerative bone changes								
Condylar degeneration	405 (20.1)		17 (28.3)		422 (20.3)	0.118†	1.57	0.9-2.8
Change in articular eminence shape	12 (0.6)		0 (0.0)		12 (0.6)	1.000†	NC	NC
Glenoid fossa degeneration	39 (1.9)		2 (3.3)		41 (2.0)	0.330†	1.7	0.4-7.4
Effusion	21 (1.0)		0 (0.0)		21 (1.0)	1.000†	NC	NC

* Chi-square test;

† Fisher's exact test. OR, odds ratio; CI, confidence interval; NC, not calculable.

As can be seen in Table 1, DDWR was significantly associated with disc deformity and with a change in the shape of the articular eminence (*p* < 0.001 for both). Of the 295 TMJs with DDWR, 130 showed one or more of the abnormalities listed in the table, whereas the remaining 165 showed none.

Table 2 shows that DDWoR was significantly associated with disc deformity, degeneration of the condyle, degeneration of the glenoid fossa, and effusion (*p* < 0.001 for all). Of the 428 TMJs with DDWoR, 350 showed one or more of the abnormalities listed in the table, whereas the remaining 78 showed none.

As shown in Table 3, posterior disc displacement was significantly associated with disc deformity (*p* < 0.001). Of the 60 TMJs with posterior disc displacement, 36 TMJs presented one or more of the abnormalities listed in the table, whereas the remaining 24 TMJs showed none.

As previously mentioned, we evaluated decreases and increases in condylar translation. Neither was found to be statistically associated with disc deformity, degenerative osseous changes, or any type of disc displacement (*p* ≥ 0.05; odds ratio < 1 for all).

## DISCUSSION

Clinical examination is insufficient to diagnose TMJ changes^(^^1^^)^. MRI is the best method for diagnosing TMD; in addition to providing excellent routine static images, it has the ability to analyze disc position and condylar excursion throughout the dynamic process of mouth opening and closing by obtaining multiple static images in series^(^^2^^,^^21^^)^.

The higher prevalence of TMDs in female patients is a well-known fact. Some authors have reported that more than 75% of affected individuals are women^(^^3^^,^^22^^,^^23^^)^, which is consistent with the gender distribution of the sample in the present study (85.8% women and 14.2% men). The 39.4% prevalence of normal MRI findings in our sample may be explained by the presence of muscular disorder alone without anatomical repercussions.

Regarding the type of disc displacement, the proportion of patients with DDWoR was higher than that of those with DDWR (20.6% vs. 14.2%). This is consistent with the findings of previous studies^(^^4^^,^^21^^)^. However, a study involving 218 symptomatic TMD patients showed a higher incidence of DDWR^(^^5^^)^, perhaps because the most common age group was 16-25 years (accounting for 37% of the sample), constituting a younger population than that evaluated in the present study, given that DDWR is more common in younger patients and represents the initial stage of TMJ abnormalities. In the present study, the prevalence of posterior disc displacement was 2.9%, corroborating its rarity in the literature^(^^1^^,^^4^^,^^24^^)^. As reported in a previous study^(^^1^^)^, we found only one case of lateral disc displacement and no cases of medial disc displacement. Lateral disc displacement and medial disc displacement are rare because the lateral and medial surfaces are supported more firmly by their ligaments, whereas anterior disc displacement follows the line of least resistance^(^^8^^)^.

The results of the present study indicate an association between disc deformity and anteroposterior disc displacement, especially DDWoR, which was associated with a higher risk of disc deformity than were the other forms of displacement (odds ratio = 5.2). That relationship has been described in the literature^(^^1^^,^^9^^,^^21^^,^^25^^,^^26^^)^, suggesting that disc deformity reflects the severity of internal derangement. According to some studies^(^^1^^,^^25^^)^, a greater degree of anterior disc displacement is associated with a higher probability of severe morphological changes in the disc, which becomes permanently displaced, losing its normal biconcave shape and becoming difficult to reduce in the condyle.

Osteoarthritis of the TMJ is a disease that typically occurs after the articular disc is displaced and bone contact is established between the condyle and the glenoid fossa. Although there are several reports in the literature, the relationship between osteoarthritis and internal derangement of the TMJ is not yet fully understood^(^^7^^,^^27^^)^.

Some studies have reported an association between DDWoR and degenerative bone changes^(^^5^^,^^9^^,^^21^^,^^23^^,^^28^^,^^29^^)^. Our results show that both types of displacement (DDWR and DDWoR) were significantly associated with degenerative bone changes. However, DDWoR was associated with degeneration of two bone structures, the condyle and the glenoid fossa, whereas DDWR was associated only with changes in the shape of the articular eminence. It has been suggested that the relationship between DDWoR and osteoarthritis is attributable to the fact that DDWoR involves a permanently displaced disc^(^^5^^)^. The disc is not located between the condyle and the articular eminence, neither during the functional state nor at rest. When the cartilage of the joints is damaged and worn due to the friction of the bones between them, degenerative changes occur in the underlying bone^(^^5^^)^.

It has been reported that a greater number of flattened condyles is associated with DDWR^(^^30^^-^^32^^)^, which suggests that this may be the initial change in a progressive disease. It has also been observed that bone changes in the condyle are statistically similar in DDWR and DDWoR, except for the combination of erosion and osteophytes, which is more common in DDWoR^(^^29^^-^^32^^)^. In the present study, 25.0% of the patients exhibited condylar degeneration, and 48.1% of those patients had DDWoR, constituting a significant association between the two. Roh et al.^(^^10^^)^ obtained similar results, reporting that DDWoR was present in 47.2% of patients with condylar degeneration, and that, although degenerative bone changes of the condyle are associated with anterior disc displacement, such changes may also occur in a joint with a normal disc position. The authors found that the disc was in a normal position in 21.1% of TMJs with condylar degeneration.

There is controversy regarding the causes of changes in the shape of the articular eminence. Changes in the configuration of this structure are considerably less common in DDWR^(^^6^^)^. However, in the present study, we found a significant association between bone changes in the articular eminence and DDWR. That association supports the hypothesis that changes in the shape of the articular eminence could precede the progression to anterior disc displacement and occur during the transition from erosion to osteophyte formation and from DDWR to DDWoR^(^^30^^)^. Nevertheless, other studies have demonstrated an association between changes in the articular eminence shape and DDWoR^(^^4^^,^^6^^,^^9^^,^^26^^,^^28^^)^, which seems to confirm another hypothesis-namely, that bony changes in the eminence are the result of a progressive internal derangement of the TMJ, which probably leads to gradual remodeling of the articular structure. Kurita et al.^(^^26^^)^ concluded that changes in the anatomy of the articular eminence may predispose to disc displacement; conversely, disc displacement can change the shape of the articular eminence.

To our knowledge, there is not enough evidence to support a relationship between changes in the glenoid fossa and the incidence of TMD^(^^8^^)^. In the present study, glenoid fossa degeneration was significantly associated with DDWoR. Paknahad et al.^(^^8^^)^ reported that changes in the glenoid fossa were more common among patients with TMD than among individuals in a control group. Conversely, in another study^(^^4^^)^, evaluating 148 TMJs, no alterations in the glenoid fossa were observed.

We found that changes in condylar translation showed no significant association with disc deformity, degenerative bone changes, or anteroposterior disc displacement. Inconsistent results have been reported by other researchers^(^^31^^,^^33^^)^, who observed an association between decreased condylar translation and DDWoR. This suggests that the anterior position of the disc could constitute mechanical interference, preventing condylar translation to the normal position in the apex of the articular eminence when the mouth is open.

TMJ effusion represents an inflammatory response to a dysfunctional condyle-disc relationship and has been associated with anterior disc displacement^(^^10^^,^^34^^)^. There is controversy in the literature regarding the type of disc displacement most frequently correlated with joint effusion^(^^1^^)^. In the present study, joint effusion correlated with DDWoR, which is in agreement with the findings of other authors^(^^10^^,^^33^^,^^34^^)^. Some of those authors have reported that the prevalence of joint effusion is higher among patients with more advanced stages of disc displacement^(^^33^^,^^34^^)^. That could support the hypothesis that disc displacement causes accumulation of intra-articular fluid, rather than the inverse.

This study has some limitations. The observational design limits the degree to which cause and effect relationships can be inferred from the associations observed. In addition, not all possible pathological changes that can occur in the TMJ were covered, because they would have fallen outside the scope of our objective. However, the large number of cases evaluated in this study can provide the most accurate assessment to date of the frequency of and association among anatomical and functional TMJ alterations in relation to the different types of disc displacement. Furthermore, there was no control group in our study, the analyses and associations having been made between symptomatic patients with and without TMD. The novelty of our study lies not only in the number of TMJs examined but also in the fact that changes in each anatomical element of the TMJ were analyzed separately, as were changes in the dynamics of condylar translation in relation to the position of the disc.

Our results suggest that DDWoR is significantly associated with degenerative bone changes and joint effusion, as well as with a high probability of disc deformity. Changes in the articular eminence appear to represent an etiological factor for the progression of internal derangements of the TMJ.
